# Integrative Analysis of Pharmacological and Non-pharmacological Interventions in Alzheimer’s Dementia

**DOI:** 10.7759/cureus.107386

**Published:** 2026-04-20

**Authors:** Digantkumar Patel, Tejas Patel, Parag N Patel

**Affiliations:** 1 Hospital Medicine, Springfield Clinic, Springfield, USA; 2 Medicine, GMERS (Gujarat Medical Education and Research Society) Medical College and Hospital, Patan, IND

**Keywords:** agitation, alzheimer’s disease, aria, caregiver interventions, cholinesterase inhibitors, cognitive stimulation therapy, dementia, donanemab, lecanemab, memantine

## Abstract

Alzheimer’s dementia is a progressive neurodegenerative disorder in which cognitive decline, neuropsychiatric symptoms, and functional dependence emerge from a long preclinical and prodromal phase. Effective care increasingly requires an integrative approach: (1) disease-modifying pharmacology for selected patients early in the symptomatic course, (2) symptomatic pharmacotherapy for cognition and behavioral symptoms when benefits outweigh harms, and (3) non-pharmacological interventions that meaningfully affect quality of life, caregiver burden, safety, and functional outcomes across all stages. Recent advances, particularly anti-amyloid monoclonal antibodies, have reshaped early Alzheimer’s treatment while raising new implementation challenges around biomarker confirmation, monitoring for amyloid-related imaging abnormalities (ARIA), and health-system capacity. Evidence also supports structured non-pharmacological strategies (e.g., cognitive stimulation, physical activity, caregiver programs, and environmental and behavioral approaches for agitation) as core therapies rather than “adjuncts.” This narrative review synthesizes the evidence base and offers a practical, stage-based framework for combining pharmacological and non-pharmacological therapies, emphasizing person-centered goals, safety, feasibility, and equity.

## Introduction and background

Alzheimer’s dementia is the most common cause of dementia worldwide and represents a growing public health challenge as populations age and longevity increases [1-3]. Characterized by progressive cognitive decline, functional impairment, and neuropsychiatric disturbances, Alzheimer’s dementia imposes substantial burdens on patients, caregivers, and health systems [1-3]. The disease follows a prolonged clinical continuum, beginning with an extended preclinical phase marked by silent neuropathological changes, advancing through mild cognitive impairment (MCI), and ultimately progressing to overt dementia with loss of independence [4-6]. This long trajectory provides multiple opportunities for intervention, yet also demands nuanced, individualized, and stage-appropriate care strategies [1,4-6].

Historically, therapeutic approaches to Alzheimer’s dementia were largely symptomatic and focused on modest improvements or stabilization of cognition and daily functioning. Pharmacological therapies such as cholinesterase inhibitors and memantine became the standard of care for decades, offering measurable but limited benefits. During this period, non-pharmacological interventions were often viewed as supportive or ancillary, primarily aimed at caregiver education, safety, and behavioral management rather than as core components of treatment. However, growing recognition of the multidimensional nature of Alzheimer’s dementia has shifted this paradigm. Cognitive decline is inseparable from behavioral symptoms, functional dependency, psychosocial stressors, and environmental influences, necessitating a more integrative and holistic approach to care.

In recent years, advances in disease biology and biomarker science have transformed the conceptual framework of Alzheimer’s dementia. The identification of amyloid and tau pathology as central features of disease pathogenesis, coupled with the ability to detect these abnormalities through cerebrospinal fluid analysis and neuroimaging, has enabled earlier and more precise diagnosis [4-6]. This progress has culminated in the development of disease-modifying therapies, particularly anti-amyloid monoclonal antibodies, which target core pathological processes rather than downstream clinical manifestations [5-11]. These therapies have introduced the possibility of altering disease trajectory in selected patients during early symptomatic stages, fundamentally reshaping expectations for Alzheimer’s treatment [7-11].

Despite this progress, disease-modifying therapies present significant clinical and ethical challenges [1,5-11]. Their benefits appear modest and are restricted to carefully selected populations, while risks such as amyloid-related imaging abnormalities require vigilant monitoring and specialized infrastructure [7-11]. Access disparities, cost considerations, and health-system capacity further complicate real-world implementation [1,3,5,6]. As a result, pharmacological innovation alone cannot address the full spectrum of needs faced by individuals living with Alzheimer’s dementia and their caregivers. Instead, these therapies must be embedded within a broader framework of care that integrates symptomatic management, non-pharmacological strategies, and person-centered goals.

Non-pharmacological interventions have emerged as essential pillars of Alzheimer’s dementia care across all disease stages [1]. Structured cognitive stimulation programs, physical activity, occupational therapy, environmental modifications, and caregiver-focused interventions have demonstrated meaningful benefits in cognition, behavior, functional independence, and quality of life. Importantly, these approaches often carry fewer risks than pharmacological therapies and can be adapted to diverse care settings, including community, outpatient, and long-term care environments. Evidence increasingly supports their role not merely as adjuncts but as foundational therapies that address domains inadequately targeted by medications alone.

Neuropsychiatric symptoms, collectively referred to as behavioral and psychological symptoms of dementia (BPSD), represent one of the most challenging aspects of Alzheimer’s care. Agitation, aggression, depression, anxiety, psychosis, sleep disturbances, and apathy affect a majority of patients at some point during the disease course and are major drivers of caregiver burden, institutionalization, and reduced quality of life. While pharmacological treatments may be necessary for severe or refractory symptoms, they are associated with well-recognized risks, particularly in older adults with frailty and comorbidities. Consequently, best practice increasingly emphasizes non-pharmacological, individualized behavioral approaches as first-line strategies, reserving medications for carefully selected scenarios where benefits clearly outweigh harms.

Caregiver involvement is central to the management of Alzheimer’s dementia and profoundly influences patient outcomes. Informal caregivers, often family members, provide the majority of day-to-day care and decision-making support. The physical, emotional, and financial toll on caregivers is substantial and frequently underrecognized in clinical practice. Structured caregiver education, skills training, psychosocial support, and respite interventions have been shown to reduce caregiver distress, improve patient outcomes, and delay institutionalization. Integrating caregiver-focused strategies into dementia care is therefore not only compassionate but also clinically and economically prudent.

The complexity of Alzheimer’s dementia necessitates a stage-based and goal-oriented approach to treatment. Early stages emphasize accurate diagnosis, counseling, risk-benefit discussions regarding disease-modifying therapies, and promotion of cognitive and physical resilience. As the disease progresses, priorities shift toward maintaining function, managing neuropsychiatric symptoms, ensuring safety, and supporting caregivers. In advanced stages, care increasingly focuses on comfort, dignity, and quality of life, often involving palliative principles. Across all stages, shared decision-making and respect for patient values and preferences are paramount.

Health equity considerations are increasingly recognized as integral to Alzheimer’s dementia care. Socioeconomic status, race, ethnicity, geographic location, and health literacy influence access to diagnostic resources, advanced therapies, and supportive services. The emergence of biomarker-dependent treatments and specialized monitoring requirements risks widening existing disparities unless deliberate efforts are made to promote equitable implementation. Non-pharmacological interventions, many of which are low-cost and community-based, offer opportunities to mitigate inequities when thoughtfully designed and disseminated.

From a health-system perspective, Alzheimer’s dementia poses escalating challenges related to workforce capacity, care coordination, and sustainability. Effective integrative care requires collaboration among primary care clinicians, neurologists, psychiatrists, geriatricians, therapists, social workers, and community organizations. Fragmented care models are poorly suited to address the longitudinal and multidimensional needs of this population. Integrated frameworks that align pharmacological and non-pharmacological strategies within coordinated care pathways are therefore essential to improving outcomes and optimizing resource utilization [1-3].

Given the rapid evolution of therapeutic options and the expanding evidence base for non-pharmacological interventions, clinicians face increasing complexity in treatment decision-making. There is a critical need for practical, synthesis-driven guidance that moves beyond siloed discussions of individual therapies and instead emphasizes how diverse interventions can be combined, sequenced, and individualized across disease stages [1-3]. Such an approach must balance efficacy, safety, feasibility, and patient-centered goals while remaining adaptable to varied clinical settings.

This narrative review seeks to address these needs by providing an integrative analysis of pharmacological and non-pharmacological interventions in Alzheimer’s dementia. Rather than positioning therapies in isolation, the review emphasizes their complementary roles within a cohesive care framework. By synthesizing current evidence and organizing it into a stage-based, clinically actionable model, this review aims to support clinicians in delivering comprehensive, person-centered, and equitable care for individuals living with Alzheimer’s dementia and those who care for them [1-3].

Methodology

This article is a narrative review intended to provide a clinically oriented synthesis of pharmacological and non-pharmacological interventions in Alzheimer’s dementia. The literature considered for this review included major clinical guidelines, randomized controlled trials, systematic reviews, meta-analyses, landmark primary studies, and relevant regulatory or society documents related to diagnosis, biomarkers, disease-modifying therapies, symptomatic pharmacotherapy, non-pharmacological interventions, BPSD, caregiver-focused interventions, and stage-based care. Sources were selected for clinical relevance, recency, and influence on contemporary practice. The evidence was synthesized narratively and organized into thematic sections and stage-based care models to support practical application. As a narrative review, this article did not use a prespecified systematic review protocol, formal study screening workflow, or formal risk-of-bias assessment, and therefore should be interpreted as a focused clinical synthesis rather than an exhaustive systematic review.

## Review

Conceptual framework: Why “integrative” care is the default in Alzheimer’s dementia

Modern Alzheimer’s dementia care is best understood as parallel management of (a) underlying biology, (b) clinical syndrome, and (c) lived experience, including safety, caregiver capacity, and health-system supports. Evidence-based guidelines emphasize person-centered assessment, goal-concordant planning, and stepped escalation of therapies (non-pharmacologic first for many problems, pharmacologic when benefits plausibly exceed harms) [1]. In parallel, prevention and risk-reduction guidance underscores that vascular and lifestyle factors remain relevant even after diagnosis, influencing frailty, delirium risk, cerebrovascular events, and functional reserve [2]. A complementary population-level perspective is provided by the 2024 Lancet Commission update, which argues that a substantial portion of dementia burden is potentially modifiable through lifespan risk-factor interventions (education, vascular risk, sensory impairment, depression, physical activity, social contact, etc.), reinforcing that Alzheimer’s outcomes are shaped by both neuropathology and environment [3].

Diagnosis, staging, and biomarkers: Expanding the “entry points” for intervention

Historically, Alzheimer’s disease was diagnosed syndromically (progressive amnestic dementia) and treated symptomatically. The National Institute on Aging and Alzheimer's Association (NIA-AA) research framework advanced a biological definition using biomarker categories (amyloid, tau, neurodegeneration: AT(N)) [4]. More recently, revised criteria further emphasize biological staging and increasingly incorporate blood-based markers as part of the evolving landscape (especially in research and in triage pathways) [5]. The Alzheimer’s Association also provides accessible summaries of these evolving diagnostic/staging frameworks, highlighting the shift from “Alzheimer’s as a clinical syndrome” to “Alzheimer’s as a biological process" [6]. Practically, this biomarker evolution matters because disease-modifying therapies now require evidence of amyloid pathology and rely on MRI monitoring, creating implementation challenges and inequities related to testing access and infrastructure [1,6].

To summarize the evolving diagnostic and biomarker-based frameworks that inform contemporary Alzheimer’s disease staging and therapeutic eligibility, Table 1 outlines key diagnostic approaches, their clinical utility, and limitations [1,4-6].

**Table 1:** Diagnostic and biomarker frameworks in Alzheimer’s disease. AD = Alzheimer’s disease; Aβ = amyloid beta; AT(N) = amyloid, tau, neurodegeneration framework; CSF = cerebrospinal fluid; NIA-AA = National Institute on Aging and Alzheimer's Association; PET = positron emission tomography.

Framework	Core components	Clinical utility	Key limitations
Syndromic clinical diagnosis	Cognitive decline with functional impairment	Widely accessible; no advanced testing required	Limited biological specificity
NIA-AA AT(N) framework	Amyloid (A), tau (T), neurodegeneration (N)	Research classification; biological staging	Not designed for routine clinical diagnosis
Revised NIA-AA criteria (2024)	Biological staging with clinical correlation	Aligns diagnosis with the disease-modifying therapy era	Requires biomarker access
CSF biomarkers	Aβ42, total tau, phosphorylated tau	High diagnostic accuracy	Invasive; limited patient acceptance
PET imaging	Amyloid and tau visualization	Required for monoclonal antibody eligibility	Cost, availability
Blood-based biomarkers	Plasma Aβ, p-tau species	Screening and triage potential	Assay variability; confirmatory testing required

Disease-modifying therapies: Anti-amyloid monoclonal antibodies and real-world integration

Lecanemab (Leqembi)

In early symptomatic Alzheimer’s disease (MCI due to Alzheimer’s disease or mild Alzheimer’s disease dementia), lecanemab demonstrated statistically significant slowing of clinical decline compared with placebo in a large phase 3 trial, while also reducing brain amyloid [7]. Translation into practice requires careful discussion of absolute benefit, monitoring burden, infusion logistics, and risk tolerance, because adverse events include infusion reactions and amyloid-related imaging abnormalities (ARIA) [7].

Regulatory materials emphasize baseline MRI and scheduled MRI monitoring during therapy to detect ARIA and guide dosing decisions [8]. Importantly, the FDA issued a 2025 communication requiring earlier MRI monitoring (between the 2nd and 3rd infusions) in addition to other scheduled MRIs, reflecting ongoing safety signal refinement as post-marketing evidence accumulates [9]. This is a key “recent development” that directly impacts real-world protocols, staffing, and patient counseling [9].

*Donanemab (Kisunla* *)*

Donanemab also demonstrated slowing of clinical decline in early symptomatic Alzheimer’s disease in a large phase 3 randomized trial [10]. FDA labeling documents ARIA risks and monitoring requirements as well as important safety warnings (including attention to hemorrhagic risk considerations and neurologic symptom evaluation) [11]. Recent FDA/label updates have also explored dosing adjustments intended to reduce ARIA risk while preserving efficacy signals, highlighting a broader trend: disease-modifying therapy is not “set and forget” - it is a monitored program of care [11].

Lessons From Other Anti-amyloid Programs: Efficacy Heterogeneity and “Biology Versus Outcomes”

Not all amyloid-targeting programs have translated into clear clinical benefit. For example, two phase 3 trials of gantenerumab in early Alzheimer’s did not demonstrate clinical benefit despite amyloid reduction, underscoring that amyloid clearance alone may be insufficient or that timing, dosing, and downstream biology matter [12]. Aducanumab’s trajectory illustrates the complexity and controversy of accelerated approval pathways, ongoing ARIA considerations, and evolving availability; FDA materials describe its indication and monitoring requirements, while later professional summaries note discontinuation of production and time-limited access [13,14]. These experiences have broadened the field’s focus toward (1) earlier intervention, (2) multimodal targets (tau, neuroinflammation, synaptic dysfunction), and (3) more scalable biomarker strategies.

Given the rapid expansion of disease-modifying therapies targeting amyloid pathology, Table 2 summarizes currently approved and investigated anti-amyloid monoclonal antibodies, their indications, clinical benefits, and monitoring considerations [7-14].

**Table 2:** Disease-modifying therapies in early Alzheimer’s disease. AD = Alzheimer’s disease; ARIA = amyloid-related imaging abnormalities; MCI = mild cognitive impairment; MRI = magnetic resonance imaging.

Agent	Therapeutic target	Indicated disease stage	Clinical benefit	Key safety concerns	Monitoring requirements
Lecanemab	Soluble and insoluble amyloid-β	MCI or mild AD dementia	Slows cognitive and functional decline	ARIA-E/H, infusion reactions	Baseline and serial MRI
Donanemab	Amyloid plaque clearance	Early symptomatic AD	Reduces disease progression	ARIA, hemorrhagic risk	Serial MRI monitoring
Aducanumab	Amyloid-β plaques	Early AD (limited availability)	Amyloid reduction; uncertain clinical benefit	ARIA	MRI surveillance
Gantenerumab	Amyloid-β	Early AD	No demonstrated clinical benefit	ARIA	MRI monitoring

Symptomatic cognitive pharmacotherapy: Where it fits in the integrative model

Cholinesterase inhibitors (ChEIs) and memantine remain widely used because they can produce modest symptomatic benefits for cognition and global function in appropriate stages, even though they do not halt disease progression. Guideline-based prescribing stresses individualized trials and ongoing reassessment [1].

A Cochrane review supports that memantine has a small beneficial effect in moderate-to-severe Alzheimer’s disease on cognition, activities, and behavioral/mood symptoms, and notes that adding memantine to established ChEI therapy can modestly reduce deterioration compared with placebo [15]. Comparative effectiveness reviews also highlight that benefits across cognitive enhancers are generally modest and must be balanced against adverse events such as bradycardia/syncope (ChEIs), dizziness/confusion (memantine), weight loss, and polypharmacy burden [16]. Combination therapy (ChEI + memantine) has been evaluated in systematic reviews; results suggest potential incremental benefit in some populations but reinforce the need for clinical monitoring and deprescribing when harms outweigh benefits [17].

Integrative Implication

Symptomatic drugs should be framed as one layer of care that is most effective when paired with structured non-pharmacological supports (exercise, cognitive stimulation, caregiver training) that directly influence daily function and safety.

While disease-modifying therapies address underlying pathology, symptomatic pharmacologic agents remain integral to clinical management. Table 3 summarizes commonly used cognitive-enhancing medications and their clinical roles [1,15-17].

**Table 3:** Symptomatic cognitive pharmacotherapies in Alzheimer’s dementia. AD = Alzheimer’s disease; ADLs = activities of daily living; ChEI = cholinesterase inhibitor; NMDA = N-methyl-D-aspartate.

Drug class	Mechanism of action	Disease stage	Expected clinical effect	Common adverse effects
Cholinesterase inhibitors	Increase acetylcholine availability	Mild-moderate AD	Modest cognitive stabilization	GI upset, bradycardia, weight loss
Memantine	NMDA receptor antagonism	Moderate-severe AD	Small benefit in cognition and ADLs	Dizziness, confusion
ChEI + memantine	Dual neurotransmitter modulation	Moderate-severe AD	Small additive benefit	Increased side-effect burden

Blood-based biomarkers and care pathways: A “systems” perspective

Because infusion therapies require biomarker confirmation and monitoring infrastructure, blood-based biomarkers are increasingly discussed as potential tools for triage, screening, and monitoring. A 2024 review summarizes the state of blood-based biomarkers (amyloid and phosphorylated tau assays) and their emerging role in research and clinical pathways, while acknowledging assay variability and implementation cautions [18]. Population-based studies continue to evaluate how plasma biomarkers predict progression across cognitive states over time, which may inform future risk stratification and monitoring strategies [19]. Consensus-style work also emphasizes that blood biomarkers are promising but must be implemented carefully with attention to clinical context, assay performance, and downstream confirmation pathways [20]. In the near term, these developments may widen access to “biological Alzheimer’s” identification, but they also raise questions about counseling, false positives, and equity if confirmatory testing capacity remains limited.

Non-pharmacological interventions: Expanding the evidence base beyond “adjunctive care”

Non-pharmacological interventions address domains that most strongly drive real-world outcomes: function, safety, sleep, neuropsychiatric symptoms, and caregiver capacity. Importantly, these therapies are also less likely to cause iatrogenic harm than sedatives and antipsychotics.

Cognitive Stimulation and Structured Cognitive/Functional Engagement

A 2023 Cochrane review found that cognitive stimulation programs probably provide small cognitive benefits for mild-to-moderate dementia and may improve communication and social interaction, with no clear evidence of harm [21]. From an integrative standpoint, cognitive stimulation is best conceptualized as “rehabilitation for cognition,” and it can be paired with functional routines (medication supports, calendars, cues) and caregiver coaching to improve translation into daily life.

Exercise and Physical Activity: Cognition, Function, and Neuropsychiatric Outcomes

Exercise interventions have been evaluated across dementia stages with mixed but generally supportive signals for physical function and some cognitive/neuropsychiatric measures depending on intervention type, intensity, and duration. Recent systematic reviews and meta-analyses support that exercise can improve activities of daily living and physical performance, and may improve aspects of cognition or global function in some studies, though heterogeneity remains substantial [22,23]. Because falls, deconditioning, sleep disruption, and delirium risk drive morbidity, exercise’s “downstream” benefits can be clinically meaningful even when cognitive scale changes are modest.

Occupational Therapy and Functional Enablement

Community occupational therapy programs aim to support independence by adapting tasks and environments, training caregivers, and building routines. Trials such as COTiD-UK evaluated functional outcomes and caregiver competence with pragmatic, community-based delivery, illustrating both the promise and implementation complexity of occupational therapy (OT) interventions at scale [24]. Economic analyses and service evaluations highlight that benefits and cost-effectiveness can vary by setting, intensity, and integration with existing services, reinforcing the need for targeted implementation rather than a one-size-fits-all model [25].

Sleep and Circadian Interventions (Light Therapy and Structured Routines)

Sleep disruption is common in Alzheimer’s dementia and worsens cognition, agitation, and caregiver burnout. Light therapy has been studied in dementia populations; a meta-analysis of randomized trials reported improvements in sleep parameters such as night awakenings and sleep quality, with small-to-moderate effects in some outcomes [26]. Other evidence syntheses suggest possible cognitive effects, though results vary by protocol and population, and effects on agitation are inconsistent [27]. Clinically, the most durable strategy is often multicomponent: daytime activity + morning light exposure + consistent routines + management of pain/nocturia + careful medication review.

Music-Based Interventions: Nuanced Evidence and Personalization

Music-based interventions are widely used due to their feasibility and acceptability. A 2025 Cochrane review summarizes the evidence base and highlights uncertainty in effects across agitation, cognition, and emotional outcomes, with variability by intervention type and study quality [28]. Mechanistic and translational discussions emphasize that individualized music (aligned to personal history/preferences) may influence stress responses and social connection, potentially helping some patients even when average effect sizes are modest [29]. Practically, music is best deployed as part of a structured BPSD plan (trigger reduction, routine, caregiver training) rather than as a standalone “fix.”

Caregiver Interventions: One of the Highest-Impact “Treatments”

Caregiver programs reduce burden and improve coping skills, which in turn reduces crisis utilization and supports home living. The REACH II randomized trial demonstrated improvements in caregiver outcomes across diverse groups and remains a cornerstone evidence base for multicomponent caregiver training [30]. Follow-on analyses and implementations (e.g., REACH VA) support real-world feasibility and potential cost implications, reinforcing that caregiver support is a legitimate, evidence-based intervention, not merely a supportive service [31].

To emphasize the breadth and clinical impact of evidence-based non-pharmacological strategies, Table 4 summarizes key interventions and their targeted outcomes in Alzheimer’s dementia care [21-31].

**Table 4:** Non-pharmacological interventions and targeted outcomes. AD = Alzheimer’s disease; ADLs = activities of daily living.

Intervention	Primary target domain	Evidence summary	Clinical impact
Cognitive stimulation therapy	Cognition, communication	Small but consistent cognitive benefit	Improves engagement and social interaction
Exercise programs	Physical function, cognition	Improves ADLs; variable cognitive effects	Reduces falls and sleep disturbance
Occupational therapy	Functional independence	Improves task performance	Delays functional decline
Light therapy	Sleep and circadian rhythm	Improves sleep continuity	May reduce agitation
Music therapy	Agitation, mood	Variable effects; individualized response	High acceptability, low risk
Caregiver training programs	Caregiver burden	Reduces stress and depression	Delays institutionalization

BPSD management: Integrating structured non-drug algorithms with judicious pharmacology

BPSD (agitation, aggression, psychosis, sleep disturbance, wandering) drives caregiver distress and institutionalization. Evidence-based practice emphasizes the following: identify triggers (pain, delirium, constipation, urinary retention, sensory impairment, overstimulation), implement environmental/behavioral strategies, and reserve medications for severe distress or danger.

A widely cited structured approach is the DICE framework (Describe, Investigate, Create, Evaluate), which emphasizes systematic assessment and iterative care planning to minimize unnecessary psychotropic exposure [32]. When medications are needed, risk counseling is essential because antipsychotics are associated with increased mortality and other serious harms in dementia populations; observational data also show elevated risks for outcomes such as stroke, pneumonia, and fracture, reinforcing the need for “lowest dose, shortest duration, frequent review" [33,34]. Professional guidance from the American Psychiatric Association recommends antipsychotics only when symptoms are severe/dangerous or cause major distress and after non-pharmacological interventions have been attempted [35].

Newer Labeled Option: Brexpiprazole for Agitation Associated With Alzheimer’s Dementia

The FDA approved brexpiprazole for agitation associated with dementia due to Alzheimer’s disease, marking a notable regulatory development in an area historically dominated by off-label prescribing [36]. Labeling retains class warnings (including increased mortality risk in elderly patients with dementia-related psychosis) and underscores the need for careful selection and monitoring [37].

Pimavanserin and Dementia-Related Psychosis (DRP)

In a randomized discontinuation trial, responders to pimavanserin had a lower risk of relapse with continuation than with discontinuation, though the trial was stopped early, and broader generalizability remains an ongoing question [38]. This evidence supports a broader theme: DRP treatment decisions must integrate symptom severity, safety risk, caregiver capacity, and careful monitoring, especially given polypharmacy concerns.

Treating “Drivers” of BPSD: Pain as a Modifiable Trigger

Pain is frequently underrecognized in dementia and can precipitate agitation. A cluster-randomized trial showed that a systematic approach to pain management reduced agitation in nursing home residents with moderate-to-severe dementia, supporting pain assessment and analgesic optimization as a core non-pharmacological strategy in BPSD algorithms [39]. Reviews further emphasize barriers to pain recognition and management in dementia care, reinforcing the need for structured protocols [40].

A structured, stepwise approach is essential for the safe and effective management of BPSD. Table 5 outlines an evidence-based management algorithm emphasizing non-pharmacological strategies [32-40].

**Table 5:** Stepwise management algorithm for behavioral and psychological symptoms of dementia (BPSD).

Step	Intervention domain	Description
1	Medical evaluation	Identify pain, infection, delirium, and medication effects
2	Environmental modification	Reduce noise, optimize lighting, and maintain routine
3	Behavioral strategies	Redirection, validation, structured activities
4	Caregiver skills training	Communication techniques, coping strategies
5	Pharmacologic therapy (if severe)	Antipsychotics or brexpiprazole at the lowest effective dose
6	Reassessment and deprescribing	Taper medications when clinically stable

Putting it together: A stage-based, domain-based “stack” of interventions

An expanded integrative model can be operationalized as a care stack.

For early symptomatic Alzheimer’s disease, confirm diagnosis (biomarkers when needed), consider disease-modifying therapy candidacy, start/optimize symptomatic cognitive meds when appropriate, and immediately implement exercise + cognitive stimulation + caregiver training + safety planning [1,7,8].

For moderate Alzheimer’s disease, prioritize function and BPSD prevention using DICE-style assessment, caregiver skills, OT/environmental modification; use memantine/ChEI selectively; reserve psychotropics for severe/dangerous symptoms with explicit taper plans [15,32,35].

For severe Alzheimer’s disease, shift toward comfort, safety, and caregiver support; minimize iatrogenic harm; aggressively treat pain, sleep disruption, constipation, and other BPSD drivers; deprescribe low-benefit drugs [1,39].

To integrate pharmacological and non-pharmacological strategies across the disease continuum, Table 6 presents a stage-based care model for Alzheimer’s dementia [1-3,30-32].

**Table 6:** Stage-based integrative care model for Alzheimer’s dementia. AD = Alzheimer’s disease; BPSD = behavioral and psychological symptoms of dementia; ChEI = cholinesterase inhibitor; CST = cognitive stimulation therapy; MCI = mild cognitive impairment; OT = occupational therapy.

Disease stage	Primary focus	Key interventions	Care goals
MCI/mild AD	Disease modification	Anti-amyloid therapy, CST, exercise	Slow progression
Moderate AD	Function and BPSD control	Memantine ± ChEI, OT, caregiver training	Maintain independence
Severe AD	Comfort and safety	Pain control, routine-based care	Preserve dignity
End-of-life	Palliative care	Symptom relief, hospice services	Quality of life

## Conclusions

An integrative approach to Alzheimer’s dementia aligns treatment with the multidimensional reality of the disease. Symptomatic cognitive drugs and newly available anti-amyloid therapies can offer benefit, particularly in early symptomatic Alzheimer’s, but they should be embedded within robust non-pharmacological care that addresses cognition, function, BPSD, safety, and caregiver capacity. In practice, the “best” intervention is rarely a single drug or program; it is a coordinated package that is staged, person-centered, and continuously reassessed for net benefit.
